# Abundance and Extracellular Release of Phytohormones in Aero‐terrestrial Microalgae (Trebouxiophyceae, Chlorophyta) As a Potential Chemical Signaling Source^1^

**DOI:** 10.1111/jpy.13032

**Published:** 2020-07-03

**Authors:** Gregor Pichler, Wolfgang Stöggl, Fabio Candotto Carniel, Lucia Muggia, Claudio Gennaro Ametrano, Andreas Holzinger, Mauro Tretiach, Ilse Kranner

**Affiliations:** ^1^ Department of Botany University of Innsbruck Sternwartestraße 15 6020 Innsbruck Austria; ^2^ Department of Life Sciences University of Trieste Via Giorgieri 10 34127 Trieste Italy; ^3^ Grainger Bioinformatics Center The Field Museum 1400 S. Lake Shore Dr. 60605 Chicago Illinois USA

**Keywords:** chlorophytes, culture, dehydration, extracellular, indole‐3‐butyric acid, light, phytohormones, stress response

## Abstract

Phytohormones are pivotal signaling compounds in higher plants, in which they exert their roles intracellularly, but are also released for cell‐to‐cell communication. In unicellular organisms, extracellularly released phytohormones can be involved in chemical crosstalk with other organisms. However, compared to higher plants, hardly any knowledge is available on the roles of phytohormones in green algae. Here, we studied phytohormone composition and extracellular release in aero‐terrestrial Trebouxiophyceae. We investigated (a) which phytohormones are produced and if they are released extracellularly, and if extracellular phytohormone levels are (b) affected by environmental stimuli, and (c) differ between lichen‐forming and non‐lichen‐forming species. Three free‐living microalgae (*Apatococcus lobatus*, *Chloroidium ellipsoideum*, and *Myrmecia bisecta*) and three lichen‐forming microalgae (*Asterochloris glomerata*, *Trebouxia decolorans*, and *Trebouxia* sp.) were studied. Algae were grown on solid media and the following cellular phytohormones were identified by ultra‐high‐performance liquid chromatography coupled with tandem mass spectrometry (UHPLC‐MS/MS): indole‐3‐acetic acid (IAA), indole‐3‐butyric acid (IBA), abscisic acid (ABA), gibberellin A_4_ (GA_4_), and zeatin (ZT). Furthermore, IAA, IBA, ABA, jasmonic acid (JA), gibberellin A_3_ (GA_3_), and GA_4_ were found to be released extracellularly. IAA and ABA were released by all six species, and IAA was the most concentrated. Phytohormone release was affected by light and water availability, especially IAA in *A. glomerata*, *Trebouxia* sp., and *C. ellipsoideum*. No clear patterns were observed between lichen‐forming and non‐lichen‐forming species. The results are envisaged to contribute valuable baseline information for further studies into the roles of phytohormones in microalgae.

**Abbreviations**
ABA: abscisic acid
AM: arbuscular mycorrhizae
BBM: Bold's Basal Medium
BRs: brassinosteroids
CKs: cytokinins
DL: dim light (20 μmol photons · m^–2^ · s^–1^)
DL + DH: dehydration under dim light
DM: dry mass
GA_3_: gibberellin A_3_
GA_4_: gibberellin A_4_
GAs: gibberellins
HL: high light (150 μmol photons · m^–2^ · s^–1^)
HL + DH: dehydration under high light
IAA: indole‐3‐acetic acid
IBA: indole‐3‐butyric acid
JA: jasmonic acid
JAs: jasmonates
LOD: limit of detection
PTFE: polytetrafluoroethylene (Teflon)
SA: salicylic acid
SLs: strigolactones
TM: *Trebouxia* medium
UHPLC‐MS/MS: ultra‐high‐performance liquid chromatography coupled with mass spectrometry
UPW: ultrapure water
ZT: zeatin

Phytohormones, including auxins, abscisic acid (ABA), salicylic acid (SA), jasmonates (JAs), gibberellins (GAs), cytokinins (CKs), ethylene, brassinosteroids (BRs), and strigolactones (SLs), are known to play key roles in development, growth, reproduction, and stress response of higher plants (Santner et al. 2009). The term “phytohormone” was defined by Went and Thimann (1937) for higher plants, and significant efforts were made to understand their roles in model plants such as *Arabidopsis thaliana* and crops especially (Grappin et al. 2000, Reinhardt et al. 2000, Nagpal et al. 2005, Miransari and Smith 2014, Umehara et al. 2015, Verma et al. 2016). Phytohormones are also produced by bacteria, cyanobacteria, green algae, and fungi (Costacurta and Vanderleyden 1995, Sergeeva et al. 2002, Tudzynski and Sharon 2002, Grube et al. 2009). For example, ABA (Hirsch et al. 1989), auxins, GAs, BRs (Stirk et al. 2013a), CKs (Stirk et al. 2013b), jasmonic acid (JA; (Fujii et al. 1997), and SA (Onofrejova et al. 2010) have been found in green algae. According to Bradley (1991) and Tarakhovskaya et al. (2007), the roles of phytohormones in algae might be similar to those in higher plants. Nevertheless, only few studies are available regarding the biological roles of phytohormones in green algae, and most of them report on responses of chlorophytes to exogenous treatment with phytohormones. For summary of the effects of exogenous treatment with IAA, ABA, CKs, and polyamines on a broad range of aquatic algae, see Bradley (1991). An involvement of ABA in response to oxidative (Hirsch et al. 1989), osmotic (Hinojosa‐Vidal et al. 2018), and alkaline (Yoshida et al. 2003) stress factors was suggested in different chlorophytes.

Green microalgae grow in aquatic (Gray et al. 2007) or periodically wet environments (Candotto Carniel et al. 2015), and there are also aero‐terrestrial members, mostly in the Trebouxiophyceae, that are even capable of surviving desiccation (Kranner et al. 2005, 2008, Holzinger and Karsten 2013, Candotto Carniel et al. 2016, Banchi et al. 2018). These organisms can release metabolites into the environment, and their secretome includes proteins, organic acids, lipids, polysaccharides, and various low‐molecular‐weight molecules (Liu et al. 2016). To the best of our knowledge, only Maršálek et al. (1992) and Mazur et al. (2001) reported on the occurrence of indole‐3‐acetic acid (IAA) and ABA in liquid growth media of four Trebouxiophyceae species, but the influence of different environmental conditions on the release of phytohormones by green algae was not tested. Indole‐3‐carbaldehyde, known as a precursor (Bandurski et al. 1995) and degradation product of IAA (Gazarian et al. 1998), was also determined in the exudates of four different isolated lichen “photobionts” (*i.e.*, lichen‐forming microalgae; but note that lichens can also have cyanobacterial photobionts), all within the genus of *Trebouxia* (Meeßen et al. 2013). In summary, our understanding of the metabolism and biological significance of phytohormones in chlorophytes is far from understood. However, phytohormones are key signaling molecules, and evidence is emerging that they could be important players in inter‐kingdom signaling, as described in the pioneering papers of Hughes and Sperandio (2008) and Xu et al. (2015).

In this study, we investigated six terrestrial microalgae in the Trebouxiophyceae. These include the free‐living algae *Apatococcus lobatus*, *Chloroidium ellipsoideum*, and *Myrmecia bisecta*. To obtain some first insights into the putative roles of phytohormones in inter‐kingdom signaling, photobionts of the lichens *Cladonia grayi*, *Xanthoria parietina*, and *Tephromela atra* were studied (i.e., *Asterochloris glomerata*, *Trebouxia decolorans*, and *Trebouxia* sp., respectively). The lichen photobionts were chosen because they are symbionts of well‐studied lichen models, and grow sufficiently fast for producing enough biomass for the intended experiments (note that lichen symbionts are notoriously slow‐growing organisms when grown in axenic cultures). The free‐living species were chosen because of their ecological preferences in conjunction with key physiological and reproductional traits (Nugari et al. 2009, Kulichová et al. 2014, Candotto Carniel et al. 2015, Bertuzzi et al. 2017): they typically occur in soil, bark, and rock micro‐habitats, they tolerate dehydration (as do the lichen photobionts) and share a cell cycle that involves the formation of autosporangia followed by the release of autospores, and rare sexual reproduction, and they also grow sufficiently fast for producing the biomass required for the intended experiments. Eight representative phytohormones were analyzed, IAA, indole‐butyric acid (IBA), ABA, SA, JA, gibberellin A_3_ (GA_3_), gibberellin A_4_ (GA_4_), and zeatin (ZT), focussing on cellular phytohormone composition and levels, and extracellular release under different environmental conditions with varying light intensities and water availability. The latter were assumed to mimic environmental conditions that have been proposed to play a role for the process of lichenization (Stocker‐Wörgötter 2001), defined as the transition from a free‐living to a symbiotic state. The following three hypotheses were tested: (a) phytohormones can be released extracellularly; (b) extracellular release can be influenced by environmental stimuli; and (c) lichen‐forming and free‐living algae secrete a different set of phytohormones.

## MATERIALS AND METHODS

### Strain identity and culture conditions

Axenic stock cultures of *Apatococcus lobatus* (SAG 2037; 18S accession number: JX169825), *Chloroidium ellipsoideum* (SAG 3.95; ITS accession number: FM946012), and *Myrmecia bisecta* (SAG 2043; ITS accession number: LC366924) were obtained from the algal culture collection of Göttingen (Sammlung von Algenkulturen Göttingen; SAG). The *Asterochloris* strain Cgr/DA1pho was kindly provided by Daniele Armaleo (Duke University, Durham, NC, USA), originally isolated from *Cladonia grayi* soredia (Armaleo and May 2009) and identified as *Asterochloris glomerata* (Skaloud and Peksa 2010). *Trebouxia* sp. and *T. decolorans* were obtained from the culture collection of the University of Trieste; the first was the same strain described by Muggia et al. (2016) as “*Trebouxia* sp.1.” The identities of *Asterochloris* Cgr/DA1pho, *T. decolorans*, and *Trebouxia* sp. were confirmed by ITS barcodes (data not shown, details available upon request). PCR reactions (annealing temperature: 55°C) were carried out using an ITS1 and ITS4 primer pair (White et al. 1990) for *A. glomerata*, and the ITS1T and ITS4T primer pair (Kroken and Taylor 2000) for *Trebouxia* spp. PCR products were visualized on 1.5% agarose gel and cleaned using Mag‐Bind^®^ TotalPure NGS magnetic beads (Omega Bio‐Tek Norcross, Georgia, USA). Clean products and reverse primers were premixed and sent to Macrogen Europe for sequencing.

To produce sufficient biomass, stock cultures were subcultured every 14–30 d, depending on species, on solid Trebouxia medium (TM; 2% agar, pH 6.9) according to Ahmadjian (1973) and placed in a growth chamber (Percival PGC‐6HO; CLF Plant Climatics GmbH, Wertingen, Germany) under dim light (DL) of 20 μmol photons · m^–2^ · s^–1^, 14:10 h light:dark regime; 20°C) according to Yoshimura et al. (2002) and Andersen (2005).

Prior to hormone analysis, cultures were transferred to sterile hydrophilic polytetrafluoroethylene (PTFE) membranes (25 mm diameter, pore size 0.45 μm, Omnipore™, Ireland), to separate algae from agar (adapted from Gustavs et al. 2016). Three membranes per petri dish were placed on 25 mL of solid TM (as above) and inoculated with 50 µL of a 400 mg · mL^−1^ algal TM suspension (fresh weight). The algae were then grown under DL and used for experiments (see below).

### Exponential growth

Exponential growth of each species was determined to ensure that algal samples taken for subsequent experiments were at the same developmental stage (Gustavs et al. 2009). Algal biomass was harvested every two to three d, transferred to 2 mL Eppendorf tubes, frozen in liquid nitrogen and stored at −20°C prior to lyophilization (Zirbus VaCO_2_; Zirbus technology GmbH, Germany) for at least 90 h as described by Bailly and Kranner (2011). Growth curves for each species were constructed using sample dry mass (DM), determined with an analytical balance (XS 105; Mettler Toledo®, Greifensee, Switzerland), to assess the exponential growth phase for each species.

### Treatments

Cultures in the exponential growth phase were exposed to four different conditions at 20°C using a 14:10 h light:dark cycle: (1) DL (as above, used as control condition); (2) high light (HL; 150 μmol photons · m^–2^ · s^–1^); (3) a dehydration–rehydration cycle (see below) under DL (DL + DH); and (d) a dehydration–rehydration cycle (see below) under HL (HL + DH).

### Light treatments

Cultures were transferred from solid TM onto solid Bold's Basal Medium (BBM, 2% agar, pH 6.8; Bold 1949), to induce a switch from mixotrophic to autotrophic growth, in 8 cm microboxes (Microboxes Junior 40 vessels Duchefa Biochemie, Haarlem, The Netherlands) equipped with a micro‐filter strip in the lid allowing gas exchange under sterile conditions (see Fig. S1 in the Supporting Information). Then, the microboxes were stored at DL and HL conditions in a growth chamber for 7 d at 20°C.

### Dehydration treatments

For dehydration–rehydration (DL + DH and HL + DH) treatments, samples were exposed to the same conditions as described for DL and HL treatments, but with a dehydration step prior to rehydration on BBM agar. Cultures were dehydrated by transferring the PTFE membranes with the cultures into sterile 9 cm petri dishes (without lid) within humidity‐proof, sealed boxes (Ensto, 300 × 300 × 132 mm) above 750 g of sterile silica gel (Rettberg, 3–5 mm diameter with a water capacity of 30%) together with a humidity sensor (EL®‐USB‐2‐LCS T°C/RH sensor). Four petri dishes per species, each containing three 25 mm membranes with algal cultures, were placed into one box. The boxes were stored in a growth chamber (as above) at DL or HL for 22 h ± 30 min to dehydrate the algal cultures to a final water content of ≤0.1 g H_2_O · g algal DM^−1^. Thereafter, filter cultures were rehydrated by transferring them carefully onto solid BBM in sterile microboxes with air exchange filters (as above), and incubated either at DL or HL for 7 d and 20°C. All equipment was either autoclaved or surface‐sterilized with 70% EtOH (VWR Chemicals, Vienna, Austria) for three times and dried under a sterile hood.

### Blank treatments

To test if phytohormones in extracellular leachates were released by algae and were no artifacts, blank samples (i.e., filters without algae) were prepared with 50 µL of liquid TM, exactly as described for DL, HL, DL + DH, and HL + DH treatments, also including a pre‐treatment of PTFE filters on TM for 6 and 18 d.

### Sample harvesting

At the end of the treatments, all cultures were visually inspected for bacterial and fungal contamination with a stereomicroscope (Olympus SZ51, Vienna, Austria). Membranes with uncontaminated algal cultures were carefully separated from agar with a spatula. Samples were taken to assess cellular phytohormones (i.e., intracellular and cell wall‐bound phytohormones) and phytohormones released from algal cells into the medium. Hereafter, “cellular” and “extracellular” phytohormones are indicated by the subscript letters “C” and “E,” respectively, after the abbreviations for the various treatments. For quantification of extracellularly released phytohormones, the agar containing the extracellular exudates was transferred into 2 mL Eppendorf tubes and immediately frozen in liquid nitrogen. For cellular phytohormone quantification, algal cultures were removed from the membranes using a spatula and placed in 2 mL Eppendorf tubes. To remove residual extracellular hormones from the outer surface of algal cells, cultures were washed with 1 mL of liquid BBM (pH 6.8). After gentle vortexing for 5 s and centrifugation at 500*g* for 2 min at 20°C (Sigma® 3‐18 KS, Sartorius AG, Göttingen, Germany), the supernatant was removed from algal biomass with a syringe. Both cellular and extracellular samples were immediately frozen in liquid nitrogen, lyophilized for approximately 90 h ± 30 min and stored at −80°C for further UHPLC‐MS/MS measurements.

### Chemicals

All chemicals used were of highest purity (HPLC or LC‐MS‐grade) and obtained from Sigma‐Aldrich or VWR Chemicals, both Vienna, Austria, unless mentioned otherwise. All equipment used for culturing, harvesting, or ultra‐high‐performance liquid chromatography‐mass spectrometry/mass spectrometry (UHPLC‐MS/MS) was rinsed three times with LC‐MS‐grade acetonitrile or ultra‐pure water (UPW), as appropriate, before use and then dried in a fume hood.

### Sample preparation for UHPLC‐MS/MS measurements

For UHPLC‐MS/MS analysis, an adapted version of the method described in Buchner et al. (2017) was used. For cellular hormone measurements, 5 mg of lyophilized algal biomass was weighed with an analytical balance (XS 105; Mettler Toledo®). 20 mg of lyophilized agar was used to determine extracellular hormone levels. Algal biomass or agar was placed in 2 mL safe‐lock Eppendorf tubes and extracted in 1.5 mL of ice‐cold acetone/water/acetic acid (80:20:1, v:v:v) after addition of 25 µL stable isotopically labeled internal standard solution (0.5 µM ABA‐d6, 0.5 µM SA‐d4) by shaking (TissueLyser II; Qiagen, Düsseldorf, Germany) at 30 Hz for 5 min using one 5 mm glass bead (pre‐cleaned with methanol) for each Eppendorf tube, followed by centrifugation at 10,000*g*, 4°C for 12 min. Supernatants were evaporated to dryness using a SpeedVac SPD111 vacuum concentrator (Thermo Fisher Scientific Inc., Waltham, MA, USA) for 1 h to remove the acetone, followed by freezing in liquid nitrogen and lyophilization for 14 h ± 5 min. The lyophilized pellet was resuspended in 75 µL of acetonitrile by vortexing for 10 s and 5 min ultra‐sonication in an ice‐cooled water bath. Then, 75 µL of UPW were added followed by vortexing for 10 s and 5 min ultra‐sonication in an ice‐cooled water bath. The extracts were filtered through 0.2 mm PTFE filters before injection into the UHPLC‐MS/MS system.

### UHPLC‐MS/MS analysis

The phytohormones ABA, SA, JA, GA_3_, GA_4_, IAA, IBA, and ZT were identified and quantified by UHPLC‐MS/MS, using an ekspert ultra LC100 UHPLC system (Eksigent, Dublin, CA, USA) coupled to a QTRAP 4500 mass spectrometer (ABSCIEX, Framingham, MA, USA). For compound separation, a reversed‐phase column (Luna Omega C18 100 Å, 50 × 2.1 mm, 1.6 µm, Phenomenex, Torrance, CA, USA) with a SecurityGuard ULTRA Cartridge (UHPLC Fully Porous C18 with 2.1 mm inner diameter) connected ahead was used. The mobile phases contained 0.1% formic acid (v/v; solvent A) and acetonitrile containing 0.1% formic acid (solvent B). Samples were injected starting with 5% solvent B followed by a gradient to 70% solvent B (5 min), rinsing with 100% solvent B (5:01 to 6 min) and equilibration to 5% solvent B (6:30 to 8 min). The injection volume was set to 5 µL, the flow rate to 0.5 mL · min^−1^, and the column temperature to 30°C. Compounds were detected by the mass spectrometer operated in positive and negative ion mode using multiple reaction monitoring (MRM). Ion spray voltage was set to 5.5 kV (pos mode) or − 4.5 kV (neg mode), gas 1 (nebulizer gas, N_2_) to 40 psi and gas 2 (heater gas, N_2_) to 50 psi at a temperature of 500°C. Both quadrupole mass analyzers were operated at unit resolution. Peaks were automatically detected based on retention time and MRM transition. Peak areas were normalized relative to the internal standards to account for variations during sample preparation and analysis. Concentrations were calculated according to the calibration curves created with authentic standards using the software Analyst 1.6.3 and MultiQuant 2.1.1 (AB SCIEX, Framingham, MA, USA).

Blank samples containing only the solid growth medium were also processed. ABA, JA, GA_3_, GA_4_, IBA, and ZT were below the limit of detection (LOD). Trace amounts of IAA slightly above the LOD were found in 6 of 32 blank samples. However, SA was detected in all blank samples (i.e., the solid growth media contained SA at concentrations above the LOD); therefore, quantification of this hormone was considered unreliable.

### Statistics

For each species and treatment (DL, HL, DL + DH, and HL + DH), six biological replicates were measured. To test data of cellular vs. released hormone levels in DL, numerical analyses were conducted with R (version 3.5.1) and RStudio (version 1.1.383). QQ plots were used to test for normal distribution. For comparison of DL_C_ (i.e., cellular phytohormone levels after treatment with DL, as explained above) and DL_E_ (i.e., extracellular phytohormone levels after treatment with DL) data, a non‐parametric two‐sided Mann–Whitney *U* Test with continuity correction was used at *P* < 0.01. Heat map visualization was performed with Morpheus software (https://software.broadinstitute.org/morpheus).

To test data for phytohormone release under different environmental stimuli (comparison of DL_E_, HL_E_, DL + DH_E_, and DH + HL_E_), two outliers were removed and the non‐parametric Kruskal–Wallis test (*P* ≤ 0.05) followed by Dunn's post‐hoc Test (*P* ≤ 0.05) with Benjamini–Hochberg correction was conducted to verify significant differences.

## RESULTS

### Assessment of phytohormone composition under DL

**Fig. 1:**
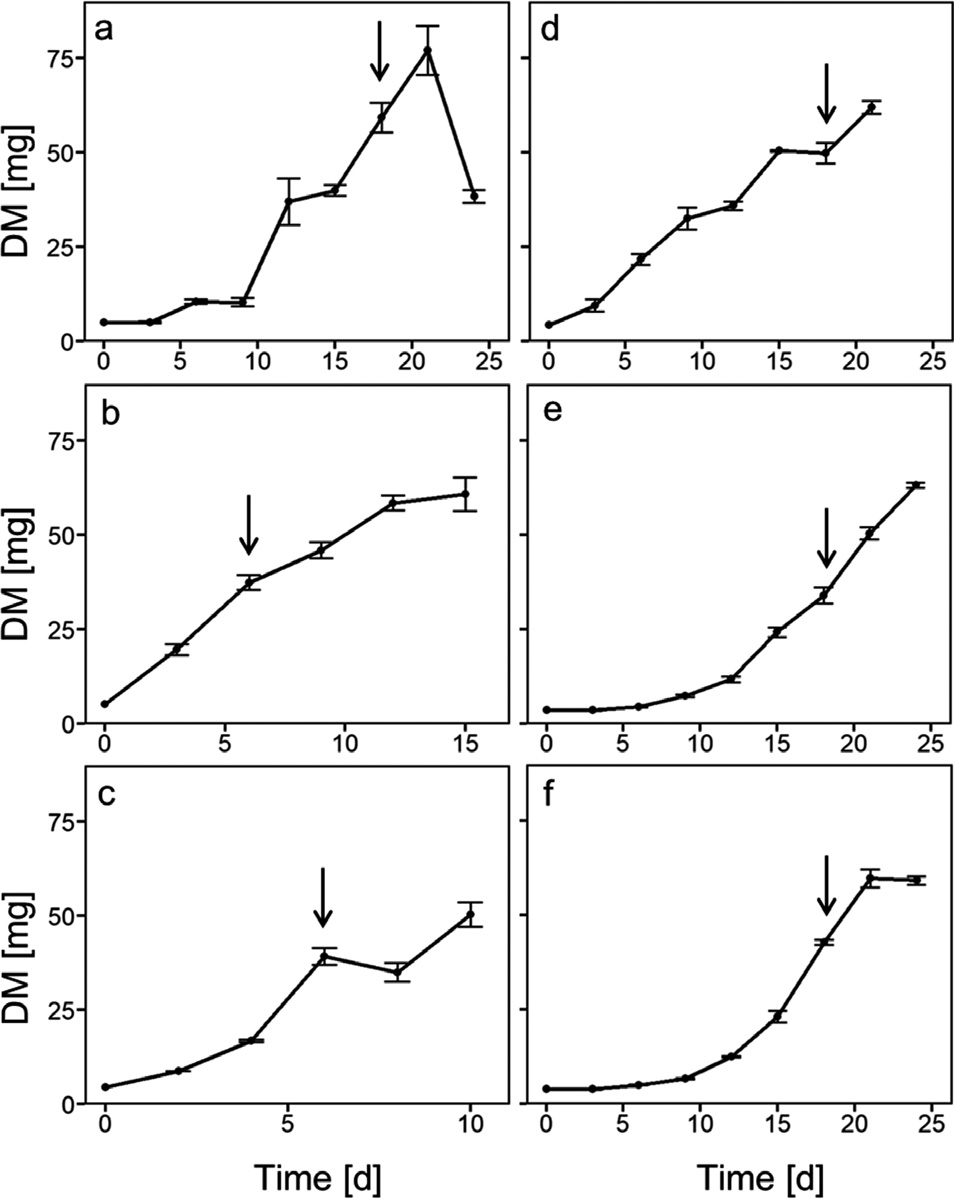
Cumulative growth of free‐living and lichen‐forming algae. Panels (a)–(c) show the free‐living algae *Apatococcus lobatus, Chloroidium ellipsoideum*, and *Myrmecia bisecta,* respectively, and panels (d)–(f) the lichen‐forming algae *Asterochloris glomerata*, *Trebouxia* sp., and *T. decolorans*, respectively. Algae were grown under DL (20 μmol photons · m^–2^ · s^–1^). Day 0 is the day of inoculation. DM refers to the dry mass of lyophilized algal material at each time interval. Data are means ± SD (*n* = 3 biological replicates). Arrows show the time points at which cultures were taken for use in subsequent experiments.

We first assessed growth rates of all algal species cultured under DL (Fig. 1), which was the control condition suitable for all six species. *Chloroidium ellipsoideum* and *Myrmecia bisecta* grew considerably faster than the other four species (Fig. 1). For subsequent experiments, cultures in a comparable growth phase were used: *C. ellipsoideum* and *M. bisecta* were taken on day 6 and *Asterochloris glomerata*, *Trebouxia decolorans*, *Trebouxia* sp., and *Apatococcus lobatus* on day 18, prior to transfer to microboxes and exposure to control conditions (DL) or treatments (HL, DL + DH, or HL + DH).

**Fig. 2:**
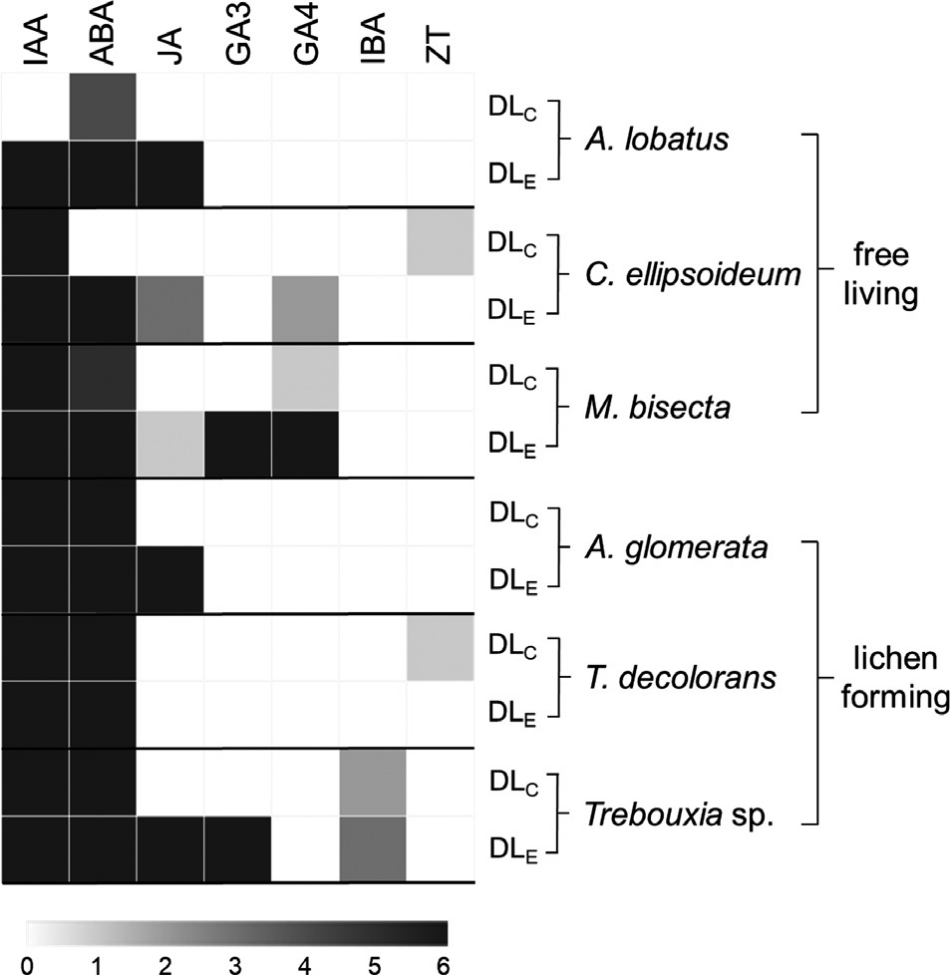
Heat map showing abundance and composition of cellular (C) and extracellular (E) phytohormones detected in free‐living and lichen‐forming algae kept under control conditions in DL (20 μmol photons · m^–2^ · s^–1^). The scale bar indicates the number of biological replicates in which an individual hormone was detected from a minimum of 0 to a maximum of 6. White fields indicate that an individual hormone was absent or below the limit of detection, and gray fields indicate low frequency.

Next, cultures in the exponential growth phase (Fig. 1) were exposed to control conditions in DL for 7 days (Fig. 2 and Table 1), and phytohormones present in algal cells and their exudates identified. Cells of the lichen‐forming alga *Trebouxia* sp. contained IAA, ABA and IBA. These three phytohormones were also released from cells, and in addition, JA and GA_3_ were found in the extracellular exudates. In *Asterochloris glomerata*, IAA and ABA were found cellularly and these two phytohormones, together with JA, were detected in the extracellular exudates. In cells of *Trebouxia decolorans*, IAA, ABA, and ZT were present, but only IAA and ABA were found extracellularly. Cells of the free‐living species *Myrmecia bisecta* contained IAA, ABA, and GA_4_, and these phytohormones were also found in the extracellular exudates together with JA and GA_3_. In *Chloroidium ellipsoideum* cells, IAA and ZT were found, and in extracellular exudates IAA, ABA, JA, and GA_4_ were detected. In cells of *A. lobatus*, only ABA was detected, which was also found in extracellular exudates together with IAA and JA (Fig. 2).

**Table 1:** Cellular and extracellular levels of the phytohormones IAA, ABA, JA, GA_3_, GA_4_, IBA, and ZT of algae after 7 d of exposure to dim light.

Species		Free‐living			Lichen‐forming		
		*A. lobatus*	*C. ellipsoideum*	*M. bisecta*	*A. glomerata*	*T. decolorans*	*Trebouxia* sp.
Phytohormone	Treatment	Mean ± SD	[nmol · g DM^−1^]				
IAA	DL_C_	≤LOD	0.11 ± 0.06	0.21 ± 0.06	0.21 ± 0.08	0.19 ± 0.05	0.20 ± 0.03
	DL_E_	*0.33 ± 0.02	*3.93 ± 0.89	*3.09 ± 0.59	*2.67 ± 0.42	*1.52 ± 0.14	*3.53 ± 0.61
ABA	DL_C_	Traces < 0.01	≤LOD	0.01 ± 0.01	0.08 ± 0.01	0.01 ± 0.00	0.02 ± 0.00
	DL_E_	*0.06 ± 0.02	*Traces < 0.01	*2.25 ± 0.39	*2.41 ± 0.32	*1.09 ± 0.29	*0.76 ± 0.11
JA	DL_C_	≤LOD	≤LOD	≤LOD	≤LOD	≤LOD	≤LOD
	DL_E_	*0.14 ± 0.08	0.01 ± 0.01	Traces < 0.01	*0.01 ± 0.00	≤LOD	*0.01 ± 0.01
GA3	DL_C_	≤LOD	≤LOD	≤LOD	≤LOD	≤LOD	≤LOD
	DL_E_	≤LOD	≤LOD	*0.16 ± 0.02	≤LOD	≤LOD	*0.10 ± 0.02
GA4	DL_C_	≤LOD	≤LOD	0.01 ± 0.01	≤LOD	≤LOD	≤LOD
	DL_E_	≤LOD	Traces < 0.01	*0.51 ± 0.06	≤LOD	≤LOD	≤LOD
IBA	DL_C_	≤LOD	≤LOD	≤LOD	≤LOD	≤LOD	0.04 ± 0.09
	DL_E_	≤LOD	≤LOD	≤LOD	≤LOD	≤LOD	Traces < 0.01
ZT	DL_C_	≤LOD	Traces < 0.01	≤LOD	≤LOD	Traces < 0.01	≤LOD
	DL_E_	≤LOD	≤LOD	≤LOD	≤LOD	≤LOD	≤LOD

Statistically significant differences (*) between cellular and extracellularly released hormones were assessed by Mann–Whitney *U* test (*P* ≤ 0.01; significances in bold font). DL, dim light; subscript letters C and E denote cellular and extracellular phytohormone levels, respectively, normalized to algal dry mass (DM).

Normalized to g algal DM, IAA levels were around 10 times higher in exudates compared to cellular levels for all species, those of ABA were between 5 and 100 times higher, and that of GA_4_, only found in *Myrmecia bisecta* cells and their exudates, was around 50 times higher. Cellular levels of JA, GA_3_, and GA_4_ were below or at the LOD in all species, but at least one of these hormones was found in exudates, at levels varying from trace amounts to high abundance (Table 1).

In summary, both IAA and ABA were present in the cells of four species studied, with the exception of *Chloroidium ellipsoideum* and *Apatococcus lobatus*, in which ABA and IAA were not detectable, respectively. Trace amounts of ZT were found in cells of *Trebouxia decolorans* and *C. ellipsoideum*, and of GA_4_ in *Myrmecia bisecta*. IAA and ABA were released by all species, and JA was released in detectable amounts by all species except *T. decolorans*. GA_3_ was detected in exudates of only two species, *Trebouxia* sp. and *M. bisecta*. GA_4_ was detected extracellularly in *M. bisecta* and *C. ellipsoideum*, and IBA was only found at levels above the LOD in cells and exudates of *Trebouxia* sp. (Fig. 2).

### Effects of high light and dehydration–rehydration treatments on extracellular phytohormone release

**Fig. 3:**
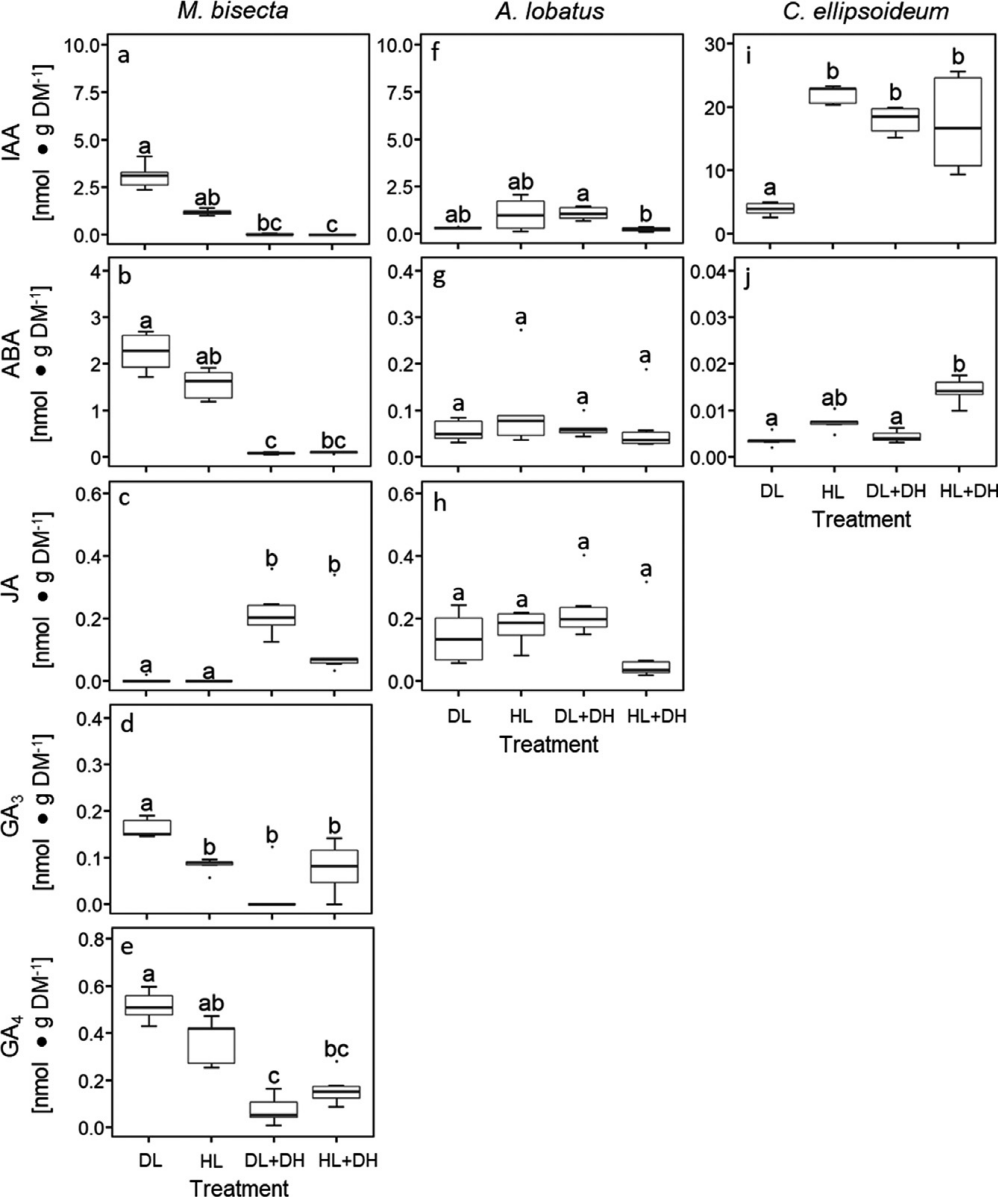
Extracellular phytohormone release of free‐living algae in response to different environmental stimuli. These included dim light (DL) used as a control, high light (HL), dehydration followed by rehydration under dim light (DL + DH), dehydration followed by rehydration under high light (HL + DH). Levels of released phytohormones of (a–e) *Myrmecia bisecta*, (f–h) *Apatococcus lobatus*, and (i and j) *Chloroidium ellipsoideum* were normalized to algal dry mass (DM). See Table S1 in the Supporting Information for further details. Boxplots (*n* = 5 to 6 biological replicates) show median, 25% and 75% percentiles, maximum and minimum values, and outliers (dots). Statistically significant differences (*P* < 0.05) are marked with different letters (Kruskal–Wallis test followed by Dunn's post‐hoc test).

High light influenced phytohormone release only in two free‐living microalgae: IAA release increased 4‐fold in *Chloroidium ellipsoideum* (Fig. 3i; Kruskal–Wallis test: chi‐squared = 15.01, df = 3, *P* < 0.05), whereas GA_3_ release by *Myrmecia bisecta* decreased (Fig. 3d; Kruskal–Wallis test: chi‐squared = 15.37, df = 3, *P* < 0.05). No significant differences (Kruskal–Wallis test: *P* > 0.05) under HL were observed for all other phytohormones and microalgae.

**Fig. 4:**
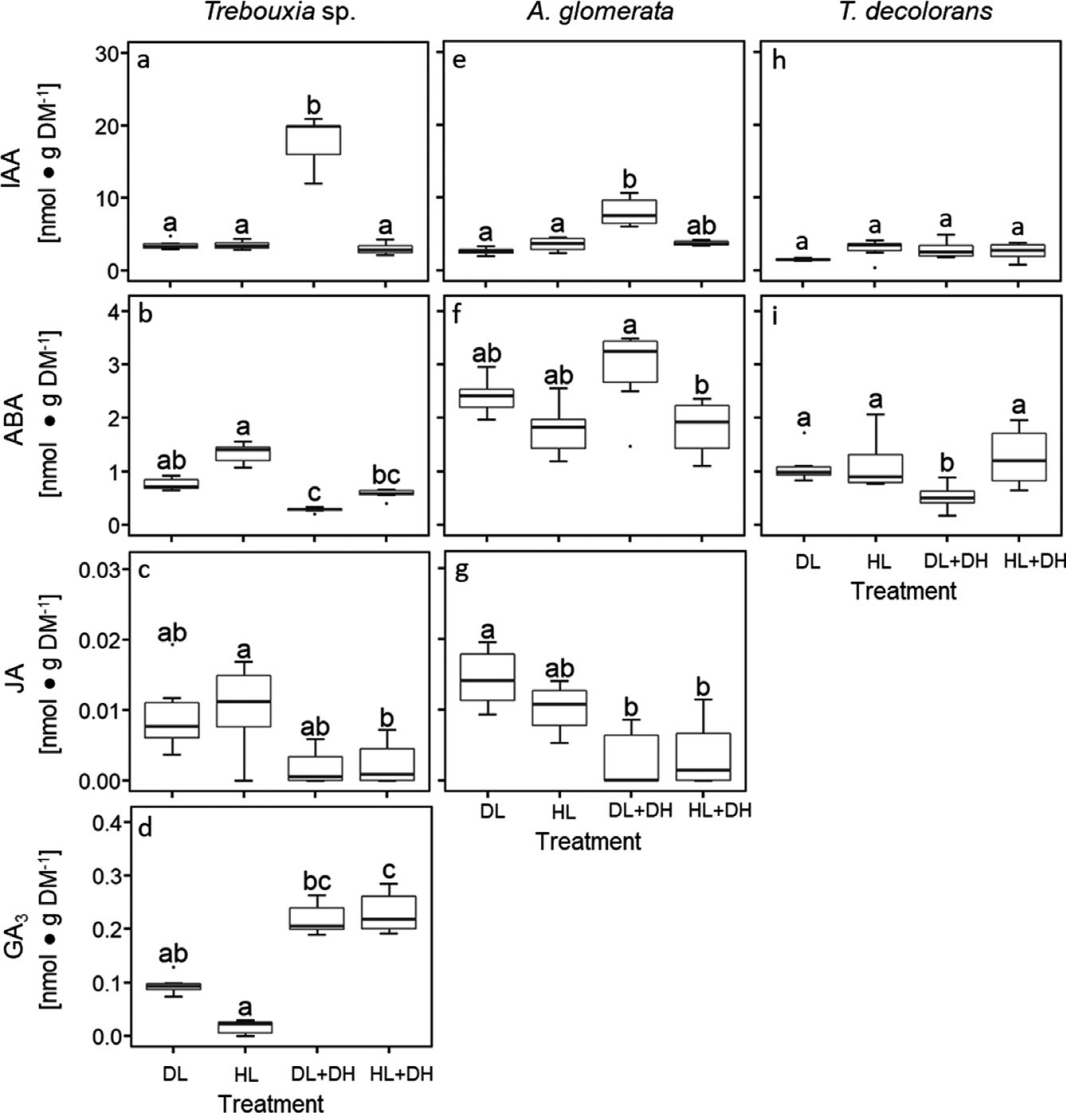
Extracellular phytohormone release by lichen‐forming algae subjected to diverse environmental stimuli. These included dim light (DL) used as control, high light (HL), dehydration followed by rehydration under dim light (DL + DH), dehydration followed by rehydration under high light (HL + DH). Levels of released phytohormones of (a–d) *Trebouxia* sp., (e–g) *Asterochloris glomerata*, and (h and i) *T. decolorans* were normalized to algal dry mass (DM). See Table S2 in the Supporting Information for further details. Boxplots (*n* = 6 biological replicates) show median, 25% and 75% percentiles, maximum and minimum values, and outliers (dots). Statistically significant differences (*P* < 0.05) are marked with different letters (Kruskal–Wallis test followed by Dunn's post‐hoc test).

Compared to DL controls, dehydration treatments (DL + DH and/or HL + DH) had the highest impact on algal phytohormone release. Both dehydration treatments (DL + DH and HL + DL), but not HL, led to bleaching of *Myrmecia bisecta* (observed visually). No bleaching was observed in the other five algae for any treatment. In response to DL + DH and HL + DH, IAA, ABA, GA_3_, and GA_4_ levels in the exudates of *M. bisecta* decreased significantly (Fig. 3, a, b, d, and e); *P* < 0.05; Kruskal–Wallis test: chi‐squared = 20.07, 18.11, 15.37, 18.32, respectively, df = 3), whereas JA levels significantly increased (Fig. 3c; Kruskal–Wallis test: chi‐squared = 19.35, df = 3; *P* < 0.05). In the lichen‐forming algae *Trebouxia* sp., *Asterochloris glomerata* (Fig. 4, a and e; Kruskal–Wallis test: chi‐squared = 16.99, 14.24, respectively, df = 3, *P* < 0.05) and in the free‐living alga *Chloroidium ellipsoideum* (Fig. 3i; Kruskal–Wallis test: chi‐squared = 15.01, df = 3, *P* < 0.05) IAA release was also strongly affected by DL + DH and/or HL + DL. Under DL + DH *A. glomerata* (Fig. 4e, Kruskal–Wallis test: chi‐squared = 16.99, df = 3, *P* < 0.05) and *Trebouxia* sp. (Fig. 4a, Kruskal–Wallis test: chi‐squared = 14.24, df = 3, *P* < 0.05) released two to four times more IAA, and *C. ellipsoideum* released, besides the previously described results for HL, four times more IAA under both, DL + DH and HL + DH (Fig. 3i, Kruskal–Wallis test: chi‐squared = 15.01, df = 3, *P* < 0.05). Furthermore, *M. bisecta* (Fig. 3b, Kruskal–Wallis test: chi‐squared = 18.11, df = 3, *P* < 0.05), *Trebouxia* sp. (Fig. 4b, Kruskal–Wallis test: chi‐squared = 21.15, df = 3, *P* < 0.05), and *T. decolorans* (Fig. 4i, Kruskal–Wallis test: chi‐squared = 9.99, df = 3, *P* < 0.05) showed decreased ABA release under DL + DH, whereas for *C. ellipsoideum* the ABA release was significantly increased under HL + DH (Fig. 3j, Kruskal–Wallis test: chi‐squared = 17.29, df = 3, *P* < 0.05). A significantly decreased JA release was found exclusively in *A. glomerata* under DL + DH and HL + DH (Fig. 4g, Kruskal–Wallis test: chi‐squared = 13.955, df = 3, *P* < 0.05). In *Trebouxia* sp., GA_3_ release strongly increased under HL + DH (Fig. 4d, Kruskal–Wallis test: chi‐squared = 19.56, df = 3, *P* < 0.05). No further significant differences (Kruskal–Wallis test: *P* > 0.05) were observed.

## DISCUSSION

The present study reports on the composition and levels of phytohormones produced and their extracellular release by free‐living and lichen‐forming Trebouxiophyceae. Furthermore, the effects of environmental stimuli, including light and dehydration treatments, on extracellular phytohormone release were tested and a potential role for phytohormones in chemical communication is discussed.

### Cellular phytohormones

Other authors reported on the occurrence of phytohormones in cells of chlorophytes, for example IAA, ABA, CKs (Lu and Xu 2015) GAs, BRs (Stirk et al. 2013a), JAs (Fujii et al. 1997), SA (Onofrejova et al. 2010), or IBA (Gupta et al. 2011). Reports on cellular phytohormones in aero‐terrestrial Trebouxiophyceae are limited to *Chlorella* sp. (see Lu and Xu 2015, for review) and *Myrmecia bisecta*, in which IAA, GAs, CKs, and BRs were found (Stirk et al. 2013a,b). With the exception of the study of Hinojosa‐Vidal et al. (2018), who found ABA in the lichen‐forming algal strain *Trebouxia* sp. TR9, we did not find reports on other phytohormones or on phytohormone composition of isolated lichen‐forming algae. In this study, we observed cellular phytohormone production (Fig. 2) in the free‐living algae *Apatococcus lobatus* (ABA), *M. bisecta* (IAA, ABA, and GA_4_), *Chloroidium ellipsoideum* (IAA and ZT), and in the lichen‐forming algae *Trebouxia* sp. (IAA, ABA, and IBA), *Asterochloris glomerata* (IAA and ABA), and *T. decolorans* (IAA, ABA, and ZT), extending the results of Stirk et al. (2013a,b) and those reviewed by Lu and Xu (2015). IAA is probably the best studied auxin in plants and also in algae, whereas IBA has received much less attention. For example, IBA was found in maize roots (Epstein et al. 1989) and in the thalli of multicellular green algae such as *Ulva* and *Monostroma* (Gupta et al. 2011), but not in unicellular free‐living or symbiotic microalgae. Epstein et al. (1986) and Ergün et al. (2002) found IAA, ABA, ZT, and GA_3_ in various lichens, for example, *Ramalina duriaei* and *Xanthoria parietina*. In these three studies, it was unclear by which symbiont these phytohormones were produced, but the fungal symbiont (i.e., the “mycobiont”) was assumed to be the main source.

### Extracellular release of phytohormones

In contrast to the cellular occurrence of phytohormones in green algae, data on the extracellular phytohormone release are scarce and limited to studying phytohormone release into liquid media (Maršálek et al., 1992, Mazur et al. 2001), whereas we used solid media. For aero‐terrestrial green algae, culture on solid growth medium is more similar to their natural environmental conditions (Ettl and Gärtner 2014). This assumption was supported by Rippin et al. (2017), who showed that physiological and transcriptional responses to dehydration of streptophytic algae grown on solid medium differ from those of algae grown in liquid medium. Phytohormones could be key molecules for inter‐kingdom signaling, as reviewed by Spaepen and Vanderleyden (2010) regarding the role of auxin in plant–microbe interactions. Xu et al. (2015) demonstrated for rice and the pathogen *Xanthomonas oryzae* that SA and ABA are involved in bidirectional cross‐communication, orchestrating host immune responses as well as modulating microbial virulence traits. Furthermore, increased extracellular IBA concentrations were proposed to play a role in arbuscular mycorrhiza formation in *Zea mays,* when co‐cultured with *Glomus intraradices* (Kaldorf and Ludwig‐Müller 2000). Hardly any knowledge exists about extracellularly released phytohormones in microalgae, but it is reasonable to assume that phytohormones could be involved in chemical crosstalk with other organisms in their environment. We observed that phytohormones were released extracellularly (Fig. 2) by the free‐living microalgae *Apatococcus lobatus* (IAA, ABA, and JA), *Chloroidium ellipsoideum* (IAA, ABA, JA, and GA_4_), *Myrmecia bisecta* (IAA, ABA, JA, GA_3_, and GA_4_), and by the lichen‐forming *Asterochloris glomerata* (IAA, ABA, and JA), *Trebouxia decolorans* (IAA and ABA), and *Trebouxia* sp. (IAA, ABA, JA, GA_3_, and IBA). Interestingly, when normalized to algal DM, the levels of phytohormones accumulated in the growth medium within 7 days were up to 100 times higher in the extracellular space than within cells (Table 1). Some phytohormones were only found in the extracellular exudates. Their rather low cellular levels, below the LOD (Fig. 2) in *A. lobatus* (IAA, JA), *C. ellipsoideum* (ABA, JA, GA_4_), *M. bisecta* (JA, GA_3_), *A. glomerata* (JA), and *Trebouxia* sp. (JA, GA_3_; i.e., suggest that these phytohormones were produced and quickly released by cells, and then accumulated in the extracellular environment). Our main interest was in studying the presence and abundance of extracellularly released phytohormones with potential roles in inter‐kingdom signaling. We appreciate that intracellularly, each phytohormone can be regulated by many mechanisms including degradation, conjugation, and interaction with other hormones, and that phytohormones present in media can be degraded by exposure to high light intensities, salinity, or pH (Dunlap and Robacker 1988, Nissen and Sutter 1990). We showed that extracellularly released phytohormones were present in the exudates, where they may represent a potential source for inter‐kingdom signaling, although it was outside the remits of this study to investigate such interactions, or how the individual phytohormones are regulated intracellularly and how they decompose after release into the extracellular space.

Furthermore, we showed that light and dehydration treatments influenced the levels of the released phytohormones (Figs. 3 and 4). No clear patterns were observed between free‐living algae and lichen‐forming algae. Instead, phytohormone release in response to environmental stimuli appeared to be species‐specific. However, common denominators are that (1) IAA and ABA were released extracellularly by all six species studied; (2) of the phytohormones studied, IAA was the most concentrated in the extracellular exudates of all six species; and (3) extracellular levels of IAA, ABA and (if found above LOD) JA were in the same order of magnitude in the three lichen‐forming algae, but strongly varied in the free‐living species. In addition, under DL + DH IAA was found in extracellular exudates at the highest levels compared to controls grown under DL in *Asterochloris glomerata* (Fig. 4e) and *Trebouxia* sp. (Fig. 4a), and under HL, DL + DH, and HL + DH in *Chloroidium ellipsoideum* (Fig. 3i). Furthermore, in *Myrmecia bisecta*, DL + DH and HL + DH treatments led to a decline in the levels of all phytohormones in the exudates, except for JA (Fig. 3c), which was found to be significantly increased by these treatments. The precise role of JA in microalgae is yet to be elucidated, but considering its involvement in programmed cell death, leaf senescence, and pathogen defence in plants (Reinbothe et al. 2009), the JA increase in response to the dehydration treatments could indicate that the viability of *M. bisecta* cells was declining, in agreement with the visually observed bleaching. By contrast, only GA_3_ levels were halved in the exudates of *M. bisecta* by the HL treatment, whereas IAA, ABA, JA, and GA_4_ did not change significantly, indicating that *M. bisecta* was likely more tolerant of HL than the two dehydration treatments (Fig. 3, a–e).

Due to the current lack of knowledge, it is difficult to draw conclusions about the potential roles of extracellularly released phytohormones in microalgae. Studies on the effects of exogenously applied phytohormones on chlorophytes may allow to offer some preliminary deductions about their putative roles. For example, Piotrowska‐Niczyporuk et al. (2012) suggested that exogenously applied phytohormones, including auxins, CKs, GAs, and JA, could be involved in the regulation of heavy metal biosorption and toxicity in *Chlorella vulgaris*. Furthermore, Piotrowska‐Niczyporuk and Bajguz (2014) showed in the same green alga that growth, pigment, and antioxidant contents can be influenced by exposure to IAA or IBA. Furthermore, exogenously applied ABA induced tolerance against oxidative stress in *Chlamydomonas reinhardtii* (Yoshida et al. 2003). In the multicellular chlorophyte *Codium fragile*, exogenous IAA treatment increased growth (Hanisak 1979). Exogenous treatment with CKs also enhanced growth of the lichen photobiont *Trebouxia irregularis* (Bačkor and Hudák 1999), and the auxins IAA and IBA increased growth of the mycobionts *Nephromopsis ornata, Myelochroa irrugans*, and *Usnea longissima* (Wang et al. 2009, Wang et al. 2010). However, care must be taken in the interpretation of data obtained from exogenously treated organisms as often very high concentrations above the physiological range are used (Bradley 1991).

## CONCLUSIONS

We used UHPLC‐MS/MS to measure cellular levels of phytohormones of six Trebouxiophyceae grown on solid medium and extracellularly released phytohormones. We found that (1) phytohormones were produced and extracellularly released by all tested algae, (2) levels of extracellular exudates were influenced by environmental stimuli, and (3) no clear patterns, neither in the composition of released phytohormones, nor in the changes of extracellular levels in response to environmental stimuli were observed between free‐living and lichen‐forming algae. Our study will help defining the physiological range of phytohormone levels to use for exogenous applications, also supporting further studies to identify the effects of phytohormones on microalgae and other organisms. More importantly, we envisage our study to be a useful basis for future studies of microalgae regarding the roles of phytohormones in molecular cross‐talk and inter‐kingdom communication, particularly with lichenising fungi and bacteria.

## Supporting information


**Figure S1**. Microboxes used to measure cellular and released phytohormone levels.

**Table S1**. Cellular and extracellular levels of the phytohormones IAA, ABA, JA, GA_3_, GA_4_, IBA and ZT of the free‐living algae, *Chloroidium ellipsoideum*, *Apatococcus lobatus* and *Myrmecia bisecta*, after 7 days of exposure to different treatments. DL, dim light; HL, high light; DL + DH, de‐rehydration cycle under dim light; HL + DH, de‐rehydration cycle under high light; subscript letters C and E denote cellular and extracellular phytohormone levels, respectively, normalized to algal dry mass (DM).

**Table S2**. Cellular and extracellular levels of the phytohormones IAA, ABA, JA, GA_3_, GA_4_, IBA and ZT of the lichen‐forming algae, *Asterochloris glomerata*, *Trebouxia decolorans* and *Trebouxia* sp., after 7 days of exposure to different treatments. DL, dim light; HL, high light; DL + DH, de‐rehydration cycle under dim light; HL + DH, de‐rehydration cycle under high light; subscript letters C and E denote cellular and extracellular phytohormone levels, respectively, normalized to algal dry mass (DM).
