# Supplementary figures and images for: Abundance and Extracellular Release of Phytohormones in Aero‐terrestrial Microalgae (Trebouxiophyceae, Chlorophyta) As a Potential Chemical Signaling Source1
Source: J Phycol. 2020 Jul 3;56(5):1295–307. doi: 10.1111/jpy.13032 (PMC7689701; doi:10.1111/jpy.13032)

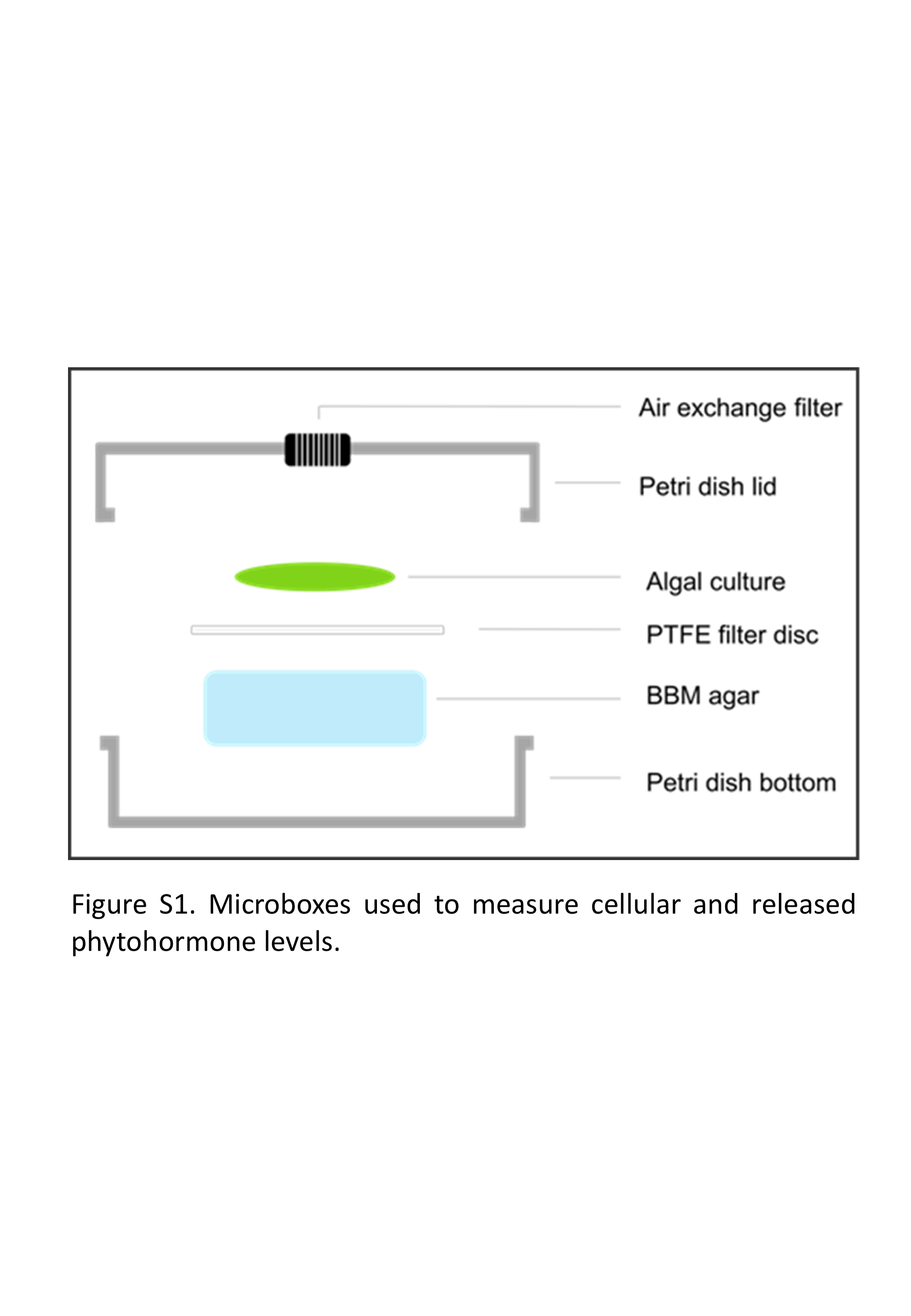

Supplement: Supplementary file 1 — Figure S1. Microboxes used to measure cellular and released phytohormone levels. [file JPY-56-1295-s001.tif]
