# Supplementary material for: Abundance and Extracellular Release of Phytohormones in Aero‐terrestrial Microalgae (Trebouxiophyceae, Chlorophyta) As a Potential Chemical Signaling Source1
Source: J Phycol. 2020 Jul 3;56(5):1295–307. doi: 10.1111/jpy.13032 (PMC7689701; doi:10.1111/jpy.13032)
Supplement: Supplementary file 2 — Table S1. Cellular and extracellular levels of the phytohormones IAA, ABA, JA, GA3, GA4, IBA and ZT of the free‐living algae, Chloroidium ellipsoideum, Apatococcus lobatus and Myrmecia bisecta, after 7 days of exposure to different treatments. DL, dim light; HL, high light; DL + DH, de‐rehydration cycle under dim light; HL + DH, de‐rehydration cycle under high light; subscript letters C and E denote cellular and extracellular phytohormone levels, respectively, normalized to algal dry mass (DM). [file JPY-56-1295-s002.docx]

**Table S1**. Cellular and extracellular levels of the phytohormones IAA, ABA, JA, GA_3_, GA_4_, IBA and ZT of the free-living algae, *C. ellipsoideum*, *A. lobatus* and *M. bisecta*, after 7 days of exposure to different treatments. DL, dim light; HL, high light; DL+DH, de-rehydration cycle under dim light; HL+DH, de-rehydration cycle under high light; subscript letters C and E denote cellular and extracellular phytohormone levels, respectively, normalized to algal dry mass (DM).

| **Species** | | *A. lobatus* | *C. ellipsoideum* | *M. bisecta* |
| --- | --- | --- | --- | --- |
| **Phytohormone**  **Treatment** | | Mean ± SD | [nmol ● g DM^-1^] |  |
| IAA [nmol ● g DW^-1^] | **DL_C_** | ≤ LOD | 0.11 ± 0.06 | 0.21 ± 0.06 |
|  | **DL_E_** | 0.33 ± 0.02 | 3.93 ± 0.89 | 3.09 ± 0.59 |
|  | **HL_E_** | 1.04 ± 0.78 | 22.01 ± 1.25 | 1.19 ± 0.14 |
|  | **DL+DH_E_** | 1.09 ± 0.31 | 17.97 ± 1.94 | 0.03 ± 0.03 |
|  | **HL+DH_E_** | 0.25 ± 0.10 | 17.38 ± 7.11 | ≤ LOD |
| ABA [nmol ● g DM^-1^] | **DL_C_** | Traces < 0.01 | ≤ LOD | 0.01 ± 0.01 |
|  | **DL_E_** | 0.06 ± 0.02 | Traces < 0.01 | 2.25 ± 0.39 |
|  | **HL_E_** | 0.10 ± 0.08 | 0.01 ± 0.00 | 1.56 ± 0.29 |
|  | **DL+DH_E_** | 0.06 ± 0.02 | Traces < 0.01 | 0.08 ± 0.02 |
|  | **HL+DH_E_** | 0.06 ± 0.06 | 0.01 ± 0.00 | 0.10 ± 0.02 |
| JA [nmol ● g DM^-1^] | **DL_C_** | ≤ LOD | ≤ LOD | ≤ LOD |
|  | **DL_E_** | 0.14 ± 0.08 | 0.01 ± 0.01 | Traces < 0.01 |
|  | **HL_E_** | 0.17 ± 0.05 | Traces < 0.01 | ≤ LOD |
|  | **DL+DH_E_** | 0.23 ± 0.08 | 0.01 ± 0.01 | 0.22 ± 0.07 |
|  | **HL+DH_E_** | 0.08 ± 0.11 | ≤ LOD | 0.11 ± 0.11 |
| GA3 [nmol ● g DM^-1^] | **DL_C_** | ≤ LOD | ≤ LOD | ≤ LOD |
|  | **DL_E_** | ≤ LOD | ≤ LOD | 0.16 ± 0.02 |
|  | **HL_E_** | ≤ LOD | ≤ LOD | 0.08 ± 0.01 |
|  | **DL+DH_E_** | ≤ LOD | ≤ LOD | 0.02 ± 0.05 |
|  | **HL+DH_E_** | ≤ LOD | ≤ LOD | 0.08 ± 0.05 |
| GA4 [nmol ● gDM^-1^] | **DL_C_** | ≤ LOD | ≤ LOD | 0.01 ± 0.01 |
|  | **DL_E_** | ≤ LOD | Traces < 0.01 | 0.51 ± 0.06 |
|  | **HL_E_** | ≤ LOD | Traces < 0.01 | 0.37 ± 0.09 |
|  | **DL+DH_E_** | ≤ LOD | Traces < 0.01 | 0.07 ± 0.05 |
|  | **HL+DH_E_** | ≤ LOD | 0.01 ± 0.01 | 0.16 ± 0.06 |
| IBA [nmol ● g DM^-1^] | **DL_C_** | ≤ LOD | ≤ LOD | ≤ LOD |
|  | **DL_E_** | ≤ LOD | ≤ LOD | ≤ LOD |
|  | **HL_E_** | Traces < 0.01 | ≤ LOD | ≤ LOD |
|  | **DL+DH_E_** | ≤ LOD | ≤ LOD | ≤ LOD |
|  | **HL+DH_E_** | ≤ LOD | ≤ LOD | ≤ LOD |
| ZT [nmol ● g DM^-1^] | **DL_C_** | ≤ LOD | Traces < 0.01 | ≤ LOD |
|  | **DL_E_** | ≤ LOD | ≤ LOD | ≤ LOD |
|  | **HL_E_** | ≤ LOD | Traces < 0.01 | ≤ LOD |
|  | **DL+DH_E_** | ≤ LOD | ≤ LOD | ≤ LOD |
|  | **HL+DH_E_** | ≤ LOD | ≤ LOD | ≤ LOD |
