# Supplementary material for: Abundance and Extracellular Release of Phytohormones in Aero‐terrestrial Microalgae (Trebouxiophyceae, Chlorophyta) As a Potential Chemical Signaling Source1
Source: J Phycol. 2020 Jul 3;56(5):1295–307. doi: 10.1111/jpy.13032 (PMC7689701; doi:10.1111/jpy.13032)
Supplement: Supplementary file 3 — Table S2. Cellular and extracellular levels of the phytohormones IAA, ABA, JA, GA3, GA4, IBA and ZT of the lichen‐forming algae, Asterochloris glomerata, Trebouxia decolorans and Trebouxia sp., after 7 days of exposure to different treatments. DL, dim light; HL, high light; DL + DH, de‐rehydration cycle under dim light; HL + DH, de‐rehydration cycle under high light; subscript letters C and E denote cellular and extracellular phytohormone levels, respectively, normalized to algal dry mass (DM). [file JPY-56-1295-s003.docx]

**Table S2**. Cellular and extracellular levels of the phytohormones IAA, ABA, JA, GA_3_, GA_4_, IBA and ZT of the lichen-forming algae, *A. glomerata*, *T. decolorans* and *Trebouxia* sp., after 7 days of exposure to different treatments. DL, dim light; HL, high light; DL+DH, de-rehydration cycle under dim light; HL+DH, de-rehydration cycle under high light; subscript letters C and E denote cellular and extracellular phytohormone levels, respectively, normalized to algal dry mass (DM).

| **Species** | | *A. glomerata* | *T. decolorans* | *Trebouxia* sp. | |
| --- | --- | --- | --- | --- | --- |
| **Phytohormone**  **Treatment** | | Mean ± SD | [nmol ● g DM^-1^] | |  |
| IAA [nmol ● g DW^-1^] | **DL_C_** | 0.21 ± 0.08 | 0.19 ± 0.05 | 0.20 ± 0.03 | |
|  | **DL_E_** | 2.67 ± 0.42 | 1.52 ± 0.14 | 3.53 ± 0.61 | |
|  | **HL_E_** | 3.61 ± 0.85 | 2.93 ± 1.25 | 3.51 ± 0.52 | |
|  | **DL+DH_E_** | 8.02 ± 1.80 | 2.89 ± 1.12 | 17.90 ± 3.30 | |
|  | **HL+DH_E_** | 3.76 ± 0.30 | 2.58 ± 1.07 | 3.02 ± 0.74 | |
| ABA [nmol ● g DM^-1^] | **DL_C_** | 0.08 ± 0.01 | 0.01 ± 0.00 | 0.02 ± 0.00 | |
|  | **DL_E_** | 2.41 ± 0.32 | 1.09 ± 0.29 | 0.76 ± 0.11 | |
|  | **HL_E_** | 1.79 ± 0.45 | 1.14 ± 0.47 | 1.34 ± 0.18 | |
|  | **DL+DH_E_** | 2.90 ± 0.72 | 0.52 ± 0.22 | 0.28 ± 0.04 | |
|  | **HL+DH_E_** | 1.82 ± 0.48 | 1.27 ± 0.50 | 0.57 ± 0.09 | |
| JA [nmol ● g DM^-1^] | **DL_C_** | ≤ LOD | ≤ LOD | ≤ LOD | |
|  | **DL_E_** | 0.01 ± 0.00 | ≤ LOD | 0.01 ± 0.01 | |
|  | **HL_E_** | 0.01 ± 0.00 | Traces < 0.01 | 0.01 ± 0.01 | |
|  | **DL+DH_E_** | Traces < 0.01 | ≤ LOD | Traces < 0.01 | |
|  | **HL+DH_E_** | Traces < 0.01 | ≤ LOD | Traces < 0.01 | |
| GA3 [nmol ● g DM^-1^] | **DL_C_** | ≤ LOD | ≤ LOD | ≤ LOD | |
|  | **DL_E_** | ≤ LOD | ≤ LOD | 0.10 ± 0.02 | |
|  | **HL_E_** | ≤ LOD | ≤ LOD | 0.02 ± 0.01 | |
|  | **DL+DH_E_** | ≤ LOD | ≤ LOD | 0.22 ± 0.03 | |
|  | **HL+DH_E_** | ≤ LOD | ≤ LOD | 0.23 ± 0.04 | |
| GA4 [nmol ● g DM^-1^] | **DL_C_** | ≤ LOD | ≤ LOD | ≤ LOD | |
|  | **DL_E_** | ≤ LOD | ≤ LOD | ≤ LOD | |
|  | **HL_E_** | ≤ LOD | ≤ LOD | ≤ LOD | |
|  | **DL+DH_E_** | ≤ LOD | ≤ LOD | ≤ LOD | |
|  | **HL+DH_E_** | ≤ LOD | ≤ LOD | ≤ LOD | |
| IBA [nmol ● g DM^-1^] | **DL_C_** | ≤ LOD | ≤ LOD | 0.04 ± 0.09 | |
|  | **DL_E_** | ≤ LOD | ≤ LOD | Traces < 0.01 | |
|  | **HL_E_** | Traces < 0.01 | ≤ LOD | Traces < 0.01 | |
|  | **DL+DH_E_** | ≤ LOD | ≤ LOD | Traces < 0.01 | |
|  | **HL+DH_E_** | Traces < 0.01 | ≤ LOD | 0.01 ± 0.00 | |
| ZT [nmol ● g DM^-1^] | **DL_C_** | ≤ LOD | Traces < 0.01 | ≤ LOD | |
|  | **DL_E_** | ≤ LOD | ≤ LOD | ≤ LOD | |
|  | **HL_E_** | ≤ LOD | ≤ LOD | ≤ LOD | |
|  | **DL+DH_E_** | ≤ LOD | ≤ LOD | ≤ LOD | |
|  | **HL+DH_E_** | ≤ LOD | ≤ LOD | ≤ LOD | |
